# Predictive value of pregnancy-associated plasma protein-A in relation to fetal loss: A systematic review and meta-analysis

**DOI:** 10.18502/ijrm.v13i6.7281

**Published:** 2020-06-30

**Authors:** Zahra Hadizadeh-Talasaz, Ali Taghipour, Seyede Houra Mousavi-Vahed, Robab Latifnejad Roudsari

**Affiliations:** ^1^Student Research Committee, Mashhad University of Medical Sciences, Mashhad, Iran.; ^2^Social Determinants of Health Research Center, Mashhad University of Medical Sciences, Mashhad, Iran.; ^3^Department of Epidemiology, School of Health, Mashhad University of Medical Sciences, Mashhad, Iran.; ^4^Department of Obstetrics and Gynecology, Faculty of Medicine, Mashhad University of Medical Sciences, Mashhad, Iran.; ^5^Nursing and Midwifery Care Research Centre, Mashhad University of Medical Sciences, Mashhad, Iran.; ^6^Department of Midwifery, School of Nursing and Midwifery, Mashhad University of Medical Sciences, Mashhad, Iran.

**Keywords:** Pregnancy-associated plasma protein-A, Fetal loss, Pregnancy, Systematic review.

## Abstract

**Background:**

For a woman with bleeding and threatened abortion, ultrasound scan is done to confirm the viability of the fetus; however, 10-15% of the embryos are eventually aborted. Distinguishing between women with good and poor prognosis can be a helpful approach.

**Objective:**

This study aimed to review the predictive value of Pregnancy-associated Plasma Protein A (PAPP-A) in relation to the diagnosis of fetal loss.

**Materials and Methods:**

The articles published in multiple databases including Web of Science, PubMed, MEDLINE, Scopus, and Persian databases such as ISC, Magiran, and IranMedx were searched for articles published until May 2019. MeSH terms was used for searching the databases including fetal loss OR pregnancy loss OR abortion OR miscarriage with the following word using AND; Pregnancy-Associated Plasma Protein-A OR PAPP-A. Two reviewers extracted data and recorded them in a pre-defined form and assessed the quality of articles using the Newcastle-Ottawa tool. Meta-analysis was done using the Comprehensive Meta-Analysis/2.0 software and MetaDisc.

**Results:**

A total number of 16 studies were eligible for the qualitative data synthesis, out of which 8 studies were included in the meta-analysis. All studies had high and medium quality. The forest plot analysis showed a sensitivity of 57% (95% CI: 53-63%), a specificity of 83% (95% CI: 80-85%), a positive likelihood ratio of 3.52 (95% CI: 2.44-5.07), a negative likelihood ratio of 0.54 (95% CI: 0.37-0.79), and a diagnostic odds ratio of 6.95 (95% CI: 3.58-13.50).

**Conclusion:**

PAPP-A cannot be recommended on a routine basis for predicting fetal loss and still further research with a combination of other biomarkers is required.

## 1. Introduction

Fetal loss is defined as the loss of the fetus before 24 wk of pregnancy, and it occurs in about 10-20% of pregnancies (1). Complex pathological factors cause fetal loss. However, the main cause of pathology of fetal loss is still in doubt. For women with bleeding and threatened abortion, ultrasound is done to confirm the viability of the fetus, however, about 10-15% of the embryos are eventually aborted (2). Once the ultrasound confirms the viability of the fetus, it is better to distinguish between women with a good prognosis and poor ones. Many studies have been done to find a marker which is able to give the prognosis. One of these markers is Pregnancy-associated Plasma Protein A (PAPP-A) (2). This protein was discovered in the blood of pregnant women in 1974 by Lin and colleagues for the first time (3), and then in multiple studies its association with adverse pregnancy outcomes was measured. Studies have shown that abnormal levels of maternal serum markers such as PAPP-A have adverse clinical outcomes in pregnancy, including fetal growth restriction, diabetes, preeclampsia, fetal death, placental abruption, miscarriage, and preterm delivery (4-7). PAPP-A is a kind of protease that plays a role in binding protein-4 (IGFBP-4) to insulin-like growth factor. Low levels of PAPP-A increase protein-4, and lead to decrease in insulin-like growth factor. Insulin-like growth factor has a key role in regulating bind of trophoblast to the decidua and low levels of it leads to abnormal implantation. As a result, abnormal amounts of PAPP-A will be a sign of abnormal placental function and may be associated with complications of pregnancy including early pregnancy loss (8, 9).

First-trimester screening is done for all pregnant women at 11-13 + 6 wk, and it is a non-invasive evaluation to determine the risk of chromosomal abnormalities, such as Down syndrome (10). Since this marker is routinely measured in all women, and it has a relation with pregnancy outcomes, this cost-effective method can be used for determining the prognosis of threatened abortion (1). Identifying patients at risk lead to increased monitoring of pregnant women who are at high risk for pregnancy complications (11). Many studies consider this marker as an important marker for abortion (12, 13). Several studies have examined the association between PAPP-A and fetal loss; however, they reported different sensitivity, specificity, and critical points and there is no same agreement among researchers; therefore, the present study discusses the results of various studies (1, 2, 8). Also, one limitation of many studies was the small sample size, which is controlled through systematic reviews. Systematic review and meta-analysis are essential tools for summarizing the evidence in an accurate, correct, and reliable way. So, researchers felt a need to conduct a systematic review to get a clear and uniform result and a comprehensive guide to clinical use.

This systematic review and meta-analysis aimed to determine the predictive value of PAPP-A for fetal loss.

## 2. Materials and Methods

### Literature search strategy and study selection

We conducted a comprehensive search through the following databases for the articles published until May 2019: Web of Science, PubMed and PubMed Central, MEDLINE, EMBASE, Scopus as well as Persian databases such as ISC (https://isc.gov.ir/en), Magiran (https://www.magiran.com/), and SID (https://www.sid.ir/Fa/Journal/). Then, a manual search was performed using references and Google scholar citations in relevant selected papers. No limitation was applied to the place, time, and language of the studies. We just had a time limitation and only the studies published between1974 and 2019 were included in our review. Lin and colleagues in 1974 discovered plasma protein-A in the blood of pregnant women for the first time, and this was the reason for choosing the date of 1974 (3). Combinations of Medical Subject Headings (MeSH) included: fetal resorption OR fetal loss OR pregnancy loss OR pregnancy failure OR abortion OR miscarriage, with the following words by using AND; Pregnancy-Associated Plasma Protein-A OR PAPP-A were used in search process. Two investigators separately searched and selected relevant articles by screening the titles and abstracts, and then full papers were retrieved for having met the inclusion criteria. Articles were included in our review if (1) they were original articles with case-control or cohort design, (2) published after 1974, (3) were conducted on human population, and (4) assessed the association between PAPP-A and fetal loss in singleton pregnancies. We excluded the review articles, papers, and abstracts presented in the conferences, letters to the editor, and articles in newspapers. At the end, a manual search through articles' references and citations of eligible articles was done in order to find articles that were not obtained by electronic searching. All papers were stored and organized in EndNote software.

### Definition of fetal loss

Although different studies have provided various definitions of fetal loss, it has been described in this review as loss of the fetus before 24 wk of pregnancy to cover all definitions (1).

### Data extraction and risk of bias assessment

Two researchers independently assessed the eligibility of the articles to extract their data and checked their qualities. Disparities during synthesis were discussed with the third investigator. Data were pulled out in a pre-defined form designed by the team. This form included the following information: 1^st^ author, year of publication, study design, the location of study, the study period, sample size in each group, the definition of fetal loss, adjusted variables, and statistical results of studies for 2 × 2 tables.

Newcastle-Ottawa tool (NOS) was used to assess the quality for cohort and case-control studies. This tool consists of three main parts: 1) selection of participants, 2) comparability, and 3) ascertainment of exposure or the outcome. Each study can obtain a maximum of nine stars. We can allocate “four stars” for part one, “two stars” for part two, and “three stars” for part three (14). The face/content validity and inter-rater reliability of the NOS has been established based on a critical review of the items by several experts (15). Scores 9, 8, and 7 were assigned to the high-quality papers 6and 5 to the moderate ones, and below 4 were assigned to the low-quality group.

### Statistical analysis

Statistical analysis was performed using the Comprehensive Meta-Analysis software (CMA version 2) and Meta-Disc. Data extracted from each study were arranged in 2 × 2 contingency tables. Then, analysis was done using 95% confidence intervals (CI) for each study. Sensitivity (true positive rate) and specificity (true negative rate) were calculated based on the reported cut-offs in the included studies and represented them with forest plots. Accordingly, false positive, true positive, false negative, and true negative values were used in the Meta-Disc. A random-effect model was used to calculate the average sensitivity, specificity, and other measures across studies. In order to show the overall accuracy of the test, SROC curve, the diagnostic odds ratio (DOR), area under the curve (AUC) and Q* were used. The likelihood ratios (LR+ and LR-) are about the percentage of patient and healthy cases that have similar test results. Summary receiver operating characteristic curve (SROC) is a useful statistical tool for assessing diagnostic efficiency of tests and evaluating the diagnostic value of variables. Since publication bias is one of the concern for meta-analysis of diagnostic studies, potential publication bias for sensitivity and specificity was evaluated using Egger's test with CMA. Subgroup analysis was not performed because of the insufficient number of studies. P < 0.05 was considered to be statistically significant.

## 3. Results

**Figure 1:**
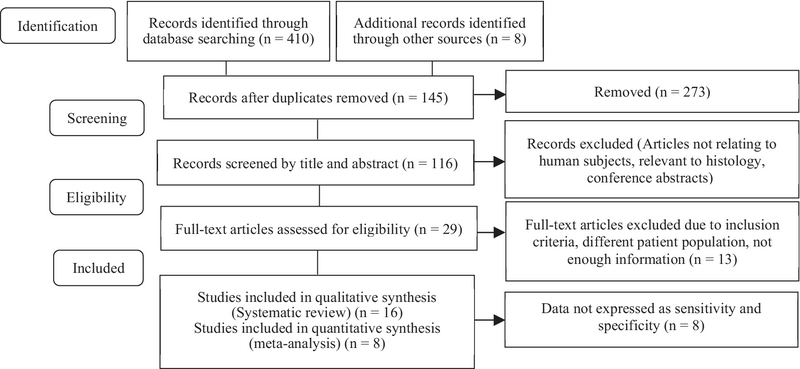
PRIZMA flow diagram for selection of eligible studies.

Figure 1 shows the process of literature search and study selection using PRISMA flowchart. The database probing identified 420 articles plus 8 more articles, which were found through searching other sources such as reference lists and citations of eligible manuscripts. By reviewing the titles and abstracts, some manuscripts were identified as duplicates (n = 273), not relating to human subjects, relevant to histology, related to stillbirth, and having a clinical trial design (n = 116), for which the full-texts were not downloaded. After reviewing full manuscripts of 29 articles in detail, 13 studies were excluded because of not meeting the inclusion criteria, enough information, or having different patient population, for example, one article was excluded because it evaluated fetal loss in the IVF population, and not in normal cases (16). Another article was not eligible because of assessing the relationship of PAPP-A and pregnancy loss in ectopic pregnancies cohort (17). Some studies evaluated stillbirth or intrauterine death (loss of the fetus after 24 wk of pregnancy). Overall 16 studies were eligible and considered relevant to be included in our systematic review, out of which 8 studies were further excluded from the meta-analysis because the data could not be extracted for the 2 × 2 tables.

The characteristics of 16 included studies are shown in Table I. All selected studies were case-control and cohort studies. They had a variety of definitions of fetal loss that ranged from 6 to 24 wk, and also had different matched variable. The statistical results extracted from each study have been demonstrated in Table II. In all articles, the mean or median of PAPP-A was lower in a group with fetal loss versus group without fetal loss. None of the studies found significant relationships between high levels of PAPP-A and adverse obstetric outcomes. Cut-offs used in various studies ranged from 0.25 to 0.66.

The quality assessment of selected articles for evaluating publication bias showed that nine studies had high quality, six had medium quality, and no study had poor quality. In total, most studies had a strong methodology. In selection domain, some articles did not have adequate case or control definition. For the comparability domain, articles that got zero had no matched variables, and if articles had adjusted variable for either in study designs or in the statistical analysis were awarded one star. In the ascertainment of exposure or assessment of outcome domain, most articles did not report non-response rate or subjects lost to follow and also used medical record only or written self-report for evaluation of exposure (Table III).

**Figure 2:**
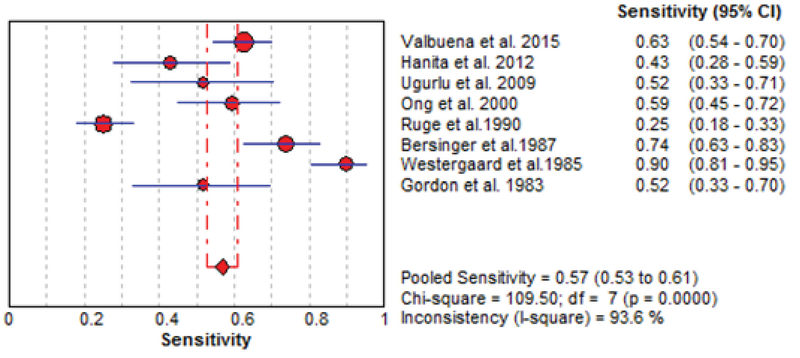
Forest plot of PAPP-A as biochemical markers for prediction of fetal loss.

**Figure 3:**
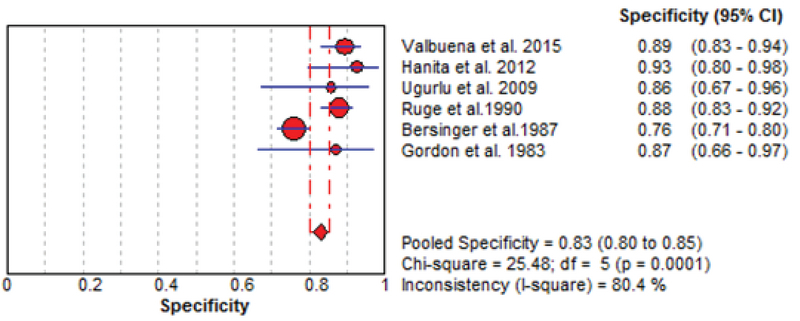
Forest plot of PAPP-A as biochemical markers for prediction of fetal loss.

**Figure 4:**
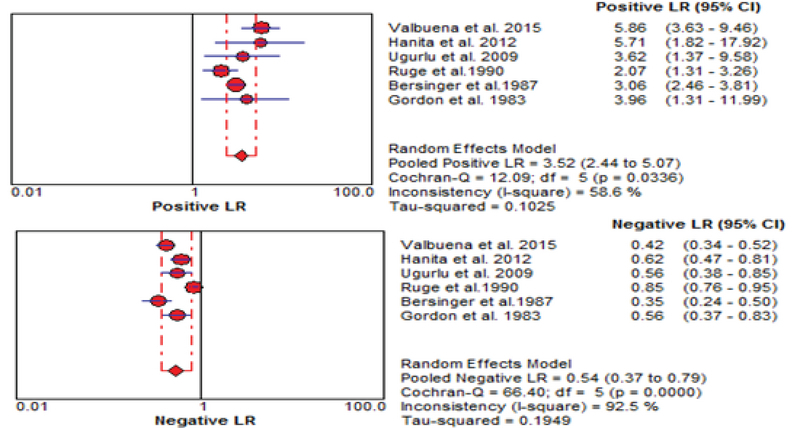
Forest plot for PAPP-A to predict fetal loss: forest plot showing likelihood ratio of a positive and negative test result with 95% confidence intervals (95% CI).

**Figure 5:**
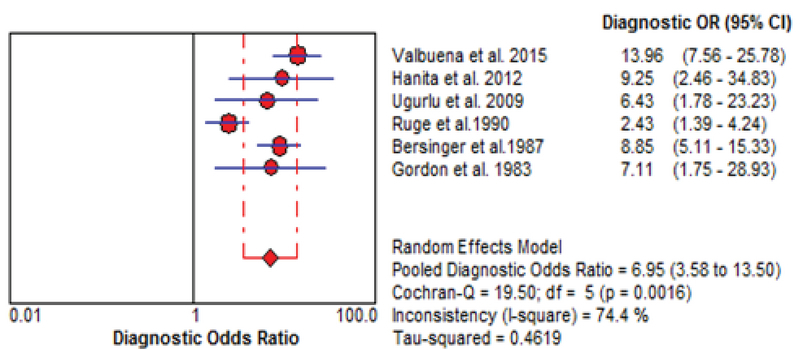
Diagnostic odds ratio forest plot for PAPP-A to predict fetal loss.

The results of the meta-analysis for PAPPA as biochemical markers to predict fetal loss are summarized in Figures 2-5. We tabulated results in a 2 × 2 contingency tables and forest plots created for the sensitivity and specificity of PAPP-A with their CI. The forest plots analysis showed a sensitivity of 57% (95% CI: 53-63%) for eight studies (1, 2, 8, 20, 24, 26, 28, 31), a specificity of 83% (95% CI: 80-85%) for six studies (1, 2, 8, 20, 28, 31), an LR+ of 3.52 (95% CI: 2.44-5.07), an LR- of 0.54 (95% CI: 0.37-0.79), and a diagnostic odds ratio of 6.95 (95% CI: 3.58-13.50).

**Figure 6:**
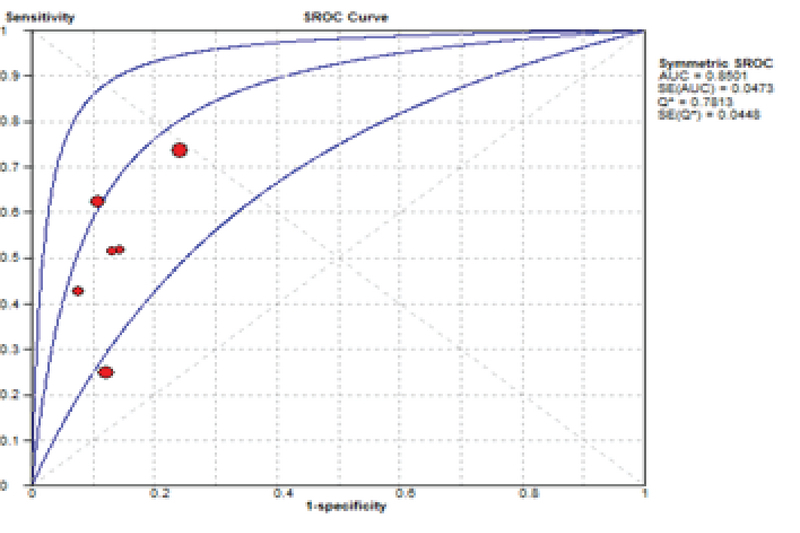
Summary receiver operating characteristic curve (SROC) for PAPP-A. Each solid circle represents each study in the meta-analysis.

“The SROC curve presents a global summary of test performance and shows the tradeoff between specificity and sensitivity. The AUC and an index Q value are discussed as useful summaries of the curve. SROC curve is shown in Figure 6. Our data showed that AUC = 0.85 and Q* = 0.78".

**Figure 7:**
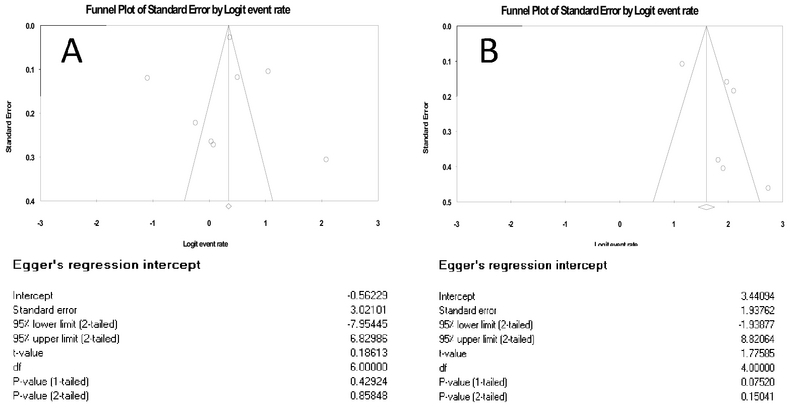
Funnel plot for the assessment of potential publication bias for sensitivity (A) and specificity (B): by Logit Event Rate and Egger's regression intercept.

Funnel plots of pooling sensitivity and specificity are demonstrated in Figure 7. The asymmetrical scattering of the points for sensitivity and specificity suggests possible publication bias which was confirmed by Egger's test intercept.

**Table 1:** Characteristics of 16 included studies


**Author/ References**	**Year**	**Study design**	**Study location**	**Study period**	**Criteria for diagnosis of fetal loss**	**Number of cases**	**Number of controls**	**Adjusted variables**
**Valbuena ** ***et al.*** ** (1)**	2015	Cohort	Spain	The 12-month period in 2011	From 8 to 13 wk	152	150	Maternal age, maternal weight, ethnicity, cigarette consumption
**Ravenswaaij ** ***et al.*** ** (18)**	2011	Historical cohort	Netherlands	July 2002 to May 2006	Pregnancy loss before 16 wk	150	- Weight
**Karim ** ***et al.*** ** (19)**	2013	Case-control	London	July 2008 and April 2010	Prior to 24 wk	256	277	Gestational age, ethnicity, smoking status, maternal weight, and IVF status
**Bersinger ** ***et al.*** ** (20)**	1987	Cohort	Switzerland	Summer 1985 and Spring 1986	Before 16 wk	80	390	_
**Hanita ** ***et al.*** ** (8)**	2012	Cohort	Malaysia	August 2010 to October 2011	Until 22 completed wk	42	40	Maternal age, gravidity, ethnic
**Spencer ** ***et al.*** ** (21)**	2006	Case-control	UK	June 1998 to December 2003	< 24 wk	230	47,770	Gestational age, maternal weight, smoking status, ethnicity
**Spencer ** ***et al.*** ** (22)**	2008	Nested case-control	Canada	- < 20 wk	77	244	Weight, smoking
**Gordon ** ***et al.*** ** (23)**	1983	Cohort	UK	_	Before 14 wk	31	23	_
**Ong ** ***et al.*** ** (24)**	2000	Cohort	UK (London)	May 1998 to 30th July 1999	< 24 completed wk	54	5297	_
**Ruge ** ***et al.*** ** (2)**	1990	Case-control	Denmark	Two-year period	Between wk 7 and wk 20	128	240	_
**Author/ References**	**Year**	**Study design**	**Study location**	**Study period**	**Criteria for diagnosis of fetal loss**	**Number of cases**	**Number of controls**	**Adjusted variables**
**Kaijomaa ** ***et al.*** ** (25)**	2017	Retrospective case-control	Finland	January 1, 2009, to December 31, 2012	Before 22 completed wk	961	961	Maternal age, maternal weight
**Westergaard ** ***et al.*** ** (26)**	1985	Cohort	Denmark	Ist January 1979 to 1980	Between 7 wk and 20 wk	77	31	_
**Barrett ** ***et al.*** ** (27)**	2008	Cohort	Australia	Two-year period	Before 19 wk and 6 day	171	10 102	_
**Ugurlu ** ***et al.*** ** (28)**	2009	Case-controlled	Turkey	January 2006 to May 2006	_	29	28	Gestational age, maternal age, gravida
**Yaron ** ***et al.*** ** (29)**	2002	Cohort	Israel	July 1998 to June 2000 (two-year )	Prior to 23 completed wk	30	1,442	Gestational age
**Tong ** ***et al.*** ** (30)**	2004	An observational study	Australia	_	At 7-13 wks' gestation	97	170	Gestational age

**Table 2:** The statistical results of the included studies


**Adjusted variable**	**Sensitivity/ Specificity**	**Cut-off**	**Diagnostic efficiency**	**AUC**	**PPV/ NPV**	**OR/RR for the outcome among patients with low PAPP-A**	**95% CI**	**Median (case)/ (control)**
**Valbuena ** ***et al.*** ** (1)**	62.1/ 89.3	0.48	72.3	0.76	67.7	- -	0.35/ 0.98
**Ravenswaaij ** ***et al.*** ** (18)**	- <0.39	- 0.78	- OR14.53	10.44-20.22	1.11/ -
**Karim ** ***et al.*** ** (19)**	- ≤0.4	- -	- RR 1.7, OR 6.2	- -
**Bersinger ** ***et al.*** ** (20)**	74.4/ 76	- 84.2	- -	- -	-
**Hanita ** ***et al.*** ** (8)**	44/ 93	0.66	- 0.66	80/86	- -	0.78/1
**Spencer ** ***et al.*** ** (21)**	- -	- -	- OR 3.25	- 0.89/ 1.04
**Spencer ** ***et al.*** ** (22)**	- ≤0.4	- -	- OR 6.2	1.92-20.03	0.4/ 0.98
**Gordon ** ***et al.*** ** (23)**	52/87	- -	- -	OR 2.2	0.3-16.6	
**Ong ** ***et al.*** ** (24)**	59.3	- -	- -	- -	0.755/ -
**Ruge ** ***et al.*** ** (2)**	25/ 87.6	- -	- -	- -	-
**Kaijomaa ** ***et al.*** ** (25)**	- 0.3	- -	- OR 7.7	- -
**Westergaard ** ***et al.*** ** (26)**	89.1	- -	- 48.8/ 98.8	RR41.8	- -
**Barrett ** ***et al.*** ** (27)**	- 0.3	- -	- RR 4.7	- 0.84/ 1.03
**Ugurlu ** ***et al.*** ** (28)**	51.8/ 86.2	- -	- -	- -	-
**Yaron ** ***et al.*** ** (29)**	- 0.25	- -	- RR 8.76	3.77-20.38	-
**Tong ** ***et al.*** ** (30)**	- -	- -	- -	- 0.14/ 1.00
AUC: Area under the curve; PPV: Positive predictive value; NPV: Negative predictive value; OR/RR: Odds ratios/relative risks; CI: C Confidence interval								

**Table 3:** Results of risk of bias based on NOS


**Patient selection**	**Patient selection**	**Comparability of groups**	**Ascertainment**	**Total NOS**
**Valbuena ** ***et al.*** ** (1)**	4	2	2	8
**Ravenswaaij ** ***et al.*** ** (18)**	3	2	2	7
**Karim ** ***et al.*** ** (19)**	4	2	3	9
**Bersinger ** ***et al.*** ** (20)**	3	0	2	5
**Hanita ** ***et al.*** ** (8)**	4	2	2	8
**Spencer ** ***et al.*** ** (21)**	3	2	2	7
**Spencer ** ***et al.*** ** (22)**	4	2	3	9
**Gordon ** ***et al.*** ** (23)**	3	1	1	5
**Ong ** ***et al.*** ** (24)**	3	1	1	5
**Ruge ** ***et al.*** ** (2)**	3	0	2	5
**Kaijomaa ** ***et al.*** ** (25)**	3	1	2	6
**Westergaard ** ***et al.*** ** (26)**	3	0	2	5
**Barrett ** ***et al.*** ** (27)**	4	0	3	7
**Ugurlu ** ***et al.*** ** (28)**	3	2	3	8
**Yaron ** ***et al.*** ** (29)**	4	1	2	7
**Tong ** ***et al.*** ** (30)**	2	1	2	5
NOS: Newcastle-Ottawa tool				

## 4. Discussion

Clinicians use diagnostic and predictive tests to identify the absence or presence of a condition in patients for the purpose of conducting an appropriate treatment plan. New diagnostic tools should be developed for improvements in ease of performance, speed, cost, patient safety, and accuracy. Systematic review of diagnostic test studies provides a summary of the accuracy of the test based on all available evidence (31). In women with pain and bleeding in the early stages of pregnancy, ultrasound alone cannot provide a definitive result for fetal loss, so it is necessary to provide an auxiliary test. This review has highlighted the predictive value of PAPP-A as biochemical markers to predict fetal loss. It is clinically important to predict the outcome of patients with bleeding and pain in the early stage of pregnancy. The prognostic value of PAPP-A was investigated in different studies and they reported different sensitivity and specificity. Our study combined the results of these studies. The result of the present study showed low predictive accuracy overall. The low predictive value may be described because of the heterogeneity between studies. Pillai and colleagues in their systematic review titled “role of serum biomarkers in the prediction of outcome in women with threatened miscarriage” found that PAPP-A had a poor and wide sensitivity ranging from 25 to 64% but a high specificity ranging from 88 to 94%. Their result about sensitivity was different from ours because our findings did not show the wide range and had 57% sensitivity (53-63), but their specificity was almost near our result of 83% (80-85). This difference may be due to the difference in the number of articles reviewed; their evaluation consisted of just three articles. Dugoff and colleagues in the FaSTER trial study showed that although a low level of PAPP-A alone associated with adverse pregnancy outcomes, it is a poor predictor of such outcomes (11).

The SROC curve summarizes the predictive power to distinguish the samples with the disease from those without the disease. It is a plot of sensitivity against specificity. The AUC is obtained from operating curve (ROC) analysis. The power considers it good if AUC is closed to 1. In the present study, the AUC was 0.85 and it shows that more than 35% of women at risk for fetal loss can be correctly classified by the predictive model (Figure 6). As seen in Table II, the results of other studies are approximately near our results, but our data showed more powerful differentiation. The diagnostic odds ratio measures the effectiveness of a diagnostic test and is less likely to change with the disease prevalence. It ranges from zero to infinity. In the present meta-analysis, we found that the mean DOR of 6.95 indicate a low level of accuracy.

LRs allow interpretation of the findings for use in clinical practice by showing how much a given test results in boost or reduce the probability of having the condition. It shows how many times sample with target disorders are more likely to receive a particular test result than those without target disorders. Our meta-analysis showed an LR+ of 3.52 (95% CI: 2.44-5.07) and an LR- of 0.54 (95% CI: 0.37- 0.79). A PLR means low maternal serum PAPP-A would be three times as likely to be seen in someone with fetal loss as opposed to someone without fetal loss. An NLR 0.5 suggests PAPP-A alone cannot detect patients with fetal loss (Figure 4).

Table I shows that the median or means values for PAPP-A was lower in fetal loss group. Zhang *et al.* reported that the level of PAPP-A mRNA in basal decidual tissue was decreased in the group who had a recurrent spontaneous abortion (32). Suzuki and colleagues said that genetic factor such as the PAPPA polymorphism may increase the risk of some types of recurrent pregnancy loss (RPL) (13). In a group of patients, Santolaya-Forgas and colleagues declared that low levels of PAPP-A concentrations can cause a down-regulation of IGF-II accessibility in fetal and placental development and that this may cause spontaneous abortions (33). Dumps and colleagues suggested that PAPP-A concentrations decrease in pregnancy failure, and circulating PAPP-A concentrations in extra uterine pregnancy (EUP) and abnormal intrauterine pregnancy (abIUP) were significantly lower in comparison to normal intrauterine pregnancy (nIUP) (p = 0.02) (17). Bischof and colleagues demonstrated that PAPP-A levels were consistently decreased or even undetectable in established ectopic pregnancies, and also after IVF when threatened abortions happen (16). The possible reason for these findings is impaired placentation because the low level of PAPP-A in maternal serum is the consequence of poor placental function that can cause fetal loss (24).

We faced some limitations in our study that must be considered. One limitation of our study was substantial heterogeneity among studies, and the sources of heterogeneity may be definition of fetal loss, the study design, adjusted variable, and gestational age at testing. Also, the publication bias could be a key concern so that our results should be interpreted with more caution (34). The other limitation was the difference in reporting statistics, which led to exclusion of some articles from being included in the meta-analysis; therefore, they made our meta-analysis limited. The other limitation was our search process. We did not search for unpublished works (including unpublished dissertations) and were limited to the Internet search. So, it is better to include unpublished papers for future studies. Finally, it should be noticed that this review was conducted on studies with singleton pregnancies and should not be generalized to all pregnant women. Also, considering the definition of fetal loss under 24 wk in this study, the results would be irrelevant to the abortions before 8-9 wk.

## 5. Conclusion

In conclusion, PAPP-A cannot be recommended for predicting fetal loss on a routine basis and still further research is required with a combination of other biomarkers. Fetal loss may be the result of a variety of etiologies and not a single disorder; therefore, a single test cannot predict all causes of fetal loss. Further studies should be conducted with a combination of different tests such as biophysical and biochemical tests to predict fetal loss with more precision.

##  Conflict of Interest

The authors declare that there is no conflict of interest.
